# Protein–like fully reversible tetramerisation and super-association of an aminocellulose

**DOI:** 10.1038/srep03861

**Published:** 2014-01-24

**Authors:** Melanie Nikolajski, Gary G. Adams, Richard B. Gillis, David Tabot Besong, Arthur J. Rowe, Thomas Heinze, Stephen E. Harding

**Affiliations:** 1 Center of Excellence for Polysaccharide Research, Institute of Organic Chemistry and Macromolecular Chemistry, Friedrich Schiller University of Jena, Humboldtstraβe 10, 07743 Jena, Germany; 2 National Centre for Macromolecular Hydrodynamics, University of Nottingham, School of Biosciences, College Road, Sutton Bonington LE12 5RD, UK

## Abstract

Unusual protein-like, partially reversible associative behaviour has recently been observed in solutions of the water soluble carbohydrates known as 6-deoxy-6-(ω-aminoalkyl)aminocelluloses, which produce controllable self-assembling films for enzyme immobilisation and other biotechnological applications. Now, for the first time, we have found a *fully reversible* self-association (tetramerisation) within this family of polysaccharides. Remarkably these carbohydrate tetramers are then seen to associate further in a regular way into supra-molecular complexes. Fully reversible oligomerisation has been hitherto completely unknown for carbohydrates and instead resembles in some respects the assembly of polypeptides and proteins like haemoglobin and its sickle cell mutation. Our traditional perceptions as to what might be considered “protein-like” and what might be considered as “carbohydrate-like” behaviour may need to be rendered more flexible, at least as far as interaction phenomena are concerned.

In a recent study^1^ protein-like, *partially* reversible associative behaviour was observed in solutions of the water soluble class of carbohydrates known as the 6-deoxy-6-(ω-aminoalkyl)aminocelluloses^2^. These molecules provide controllable self-assembling films for enzyme immobilisation and other biotechnological applications^2^ and are a class of cellulose based carbohydrate polymer which have been rendered aqueous soluble by substitution at the C-6 carbon with a particular amino chain (Fig. 1a). At C-2 there may be a further substitution of the –OH moieties with a tosyl (benzoyl) residue, e.g. 6-deoxy-6-(2-aminoethyl)aminocelluloses^2^ (AEA) - with an average degree of substitution of the amino substituent DS_Amine_ = 0.83 at C-6 (as determined by elemental analysis and nuclear magnetic resonance spectroscopy^2^) and the average degree of substitution of tosyl (benzoyl like) moieties at C-2/C-3 DS_Tosyl_ = 0.2. The powerful analytical methods of sedimentation velocity and sedimentation equilibrium in the analytical ultracentrifuge^3^ were used to demonstrate and evaluate the oligomeric nature of the preparation^4^,^5^. These methods – highly successful in the study of oligomeric behavior of proteins provide separation of species of different molecular mass without the need for any separation media – as required by chromatographic or membrane based procedures, and are not affected by supramolecular contamination as in light scattering based procedures. They also allow complete control of the concentrations of species present, vital for the consideration of reversibility phenomena. All the 6-deoxy-6-(2-aminoethyl)aminocelluloses showed regular oligomerization with at least partial reversibility^1^.

We now show for the first time a *fully reversible* self-association (tetramerisation) within this family of polysaccharides. Once formed these carbohydrate tetramers are then seen to associate further in a regular way into larger supra-molecular complexes.

## Results

**Figure 1:**
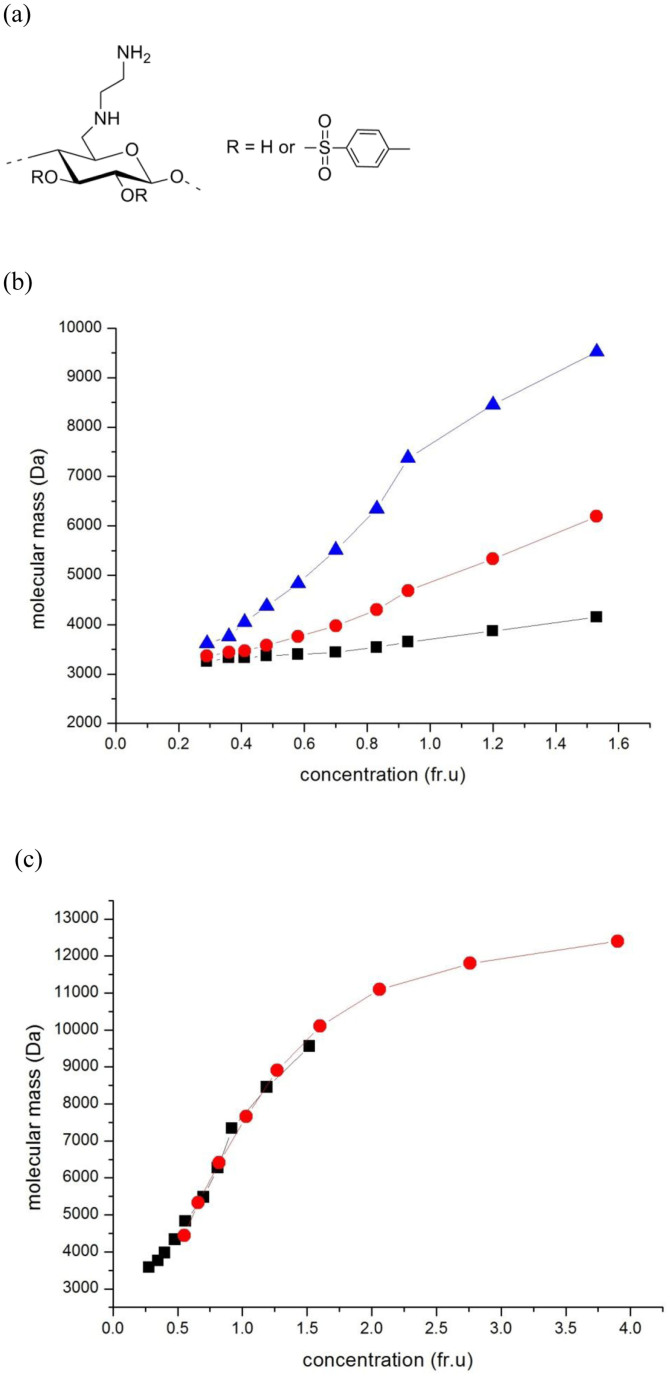
The 6-deoxy-6-(*ω*-aminoalkyl)aminocellulose AEA-1 and diagnostic ultracentrifuge plots confirming a completely reversible self-association obtained at a rotor speed of 40000 rpm and temperature 20.0°C. (a) AEA-1 repeat unit^2^ with R = H or a tosyl residue (b) Plot of molecular mass change with concentration showing a reversible self-association. The plot is of the number, weight and z-average molecular mass versus local concentration (fringe increment units) in the ultracentrifuge cell for an initial loading concentration c = 0.4 mg/ml. Black squares: n-average molecular masses; red circles, mass average molecular masses, blue triangles, z-average molecular masses. The convergence of these to a single value, the “monomer” M_1_ = 3250 Da as the concentration → 0 is a necessary diagnostic for a completely reversible association, as commonly seem in proteins. (c) Molecular mass (z-average) plotted against local concentration in the ultracentrifuge cell for two different loading concentrations: black squares c = 0.4 mg/ml, red circles c = 2.0 mg/ml. For a completely reversible self-association the plots should lie on the same curve, which they do, a feature seen for reversible protein association^6^,^7^,^8^,^9^ (see Supplementary Information).

As we found with other 6-deoxy-6-(*ω*-aminoalkyl)aminocelluloses^1^, the technique of sedimentation velocity in the analytical ultracentrifuge revealed several components present: the components sedimenting above 1 Svedberg (S = 10^−13^ sec, the same unit which is traditionally used, for example, to classify ribosome sizes) follow a step-wise series rule for globular macromolecules: s_i_ varies as i^2/3^, where i is the peak number. There appears consistently in these substances an additional peak however below 1 S at ≈0.5 S, which does not conform to this rule, and we wanted to find out its relation with the next peak in the series. To do this, a separate type of measurement in the analytical ultracentrifuge was used, namely a sedimentation equilibrium experiment^3^ (in the same instrumentation, involving an equilibration between sedimentation and diffusive forces), in order to ascertain (1) the molecular mass in Daltons (or equivalently the molar mass in g/mol) of the lowest molecular mass species in the solution (M_1_); (2) its associative properties and (3) the degree of reversibility of any self-association present. A high rotor speed was chosen (40,000 rev/min) – corresponding to a gravitation field of 125,000 g – so as to ensure the higher sedimenting components (with larger Svedberg or “S” values) were removed from optical registration and only the first two were being considered. The findings are shown in Figure 1, in terms of the average molecular masses, and how they change with the local concentration in an ultracentrifuge cell. Because of redistribution under the centrifugal field, the lowest molecular mass components (if present) will tend to predominate towards the air/solution meniscus, whereas the higher molecular mass components (if present) will become more dominant as the base of the ultracentrifuge cell (i.e. that part furthest from the centre of rotation) is approached.

From Figure 1b it is absolutely clear that the different types of average molecular masses (the number average – averaged over the number concentrations of molecules of particular masses, the weight average – average over the weight concentrations, and the z-average) converge to a single value, the (monomer molecular mass M_1_ = 3250 Daltons) at low concentration. At higher concentrations higher average molecular masses increasing towards the expected value for a tetramer (M = 13000 Da) are observed (Figure 1c). Convergence to a single value as an infinitely small concentration is approached is only compatible with the presence of a fully reversible equilibrium^6^,^7^,^8^. It also means we are considering the self-association of a near-monodisperse material. The conclusion of a fully reversible equilibrium is strongly reinforced by a plot of z-average molecular mass (chosen because it is more sensitive to mass change compared with the other averages) versus loading concentration for different loading concentrations (Figure 1c): similar overlap is obtained for the weight and number averages. For a truly reversible system the two profiles must overlay^6^,^7^ and (allowing for noise at lower concentrations) this appears to be the case. This behavior is normally seen only with proteins^8^, and has not been seen before for a carbohydrate. For comparison, data for a known reversibly dimerizing protein system –the electron transfer flavoprotein is provided in Figure S1 Supplementary Information, alongside an example for a mixture of non-interacting species – a human mucin glycoprotein (Figure S2). Other examples for protein reversible association can be seen in Teller^8^, and more recently in Harding & Rowe^9^.

We can now build a model to representative the two-stage associative behaviour of this carbohydrate.

**Figure 2:**
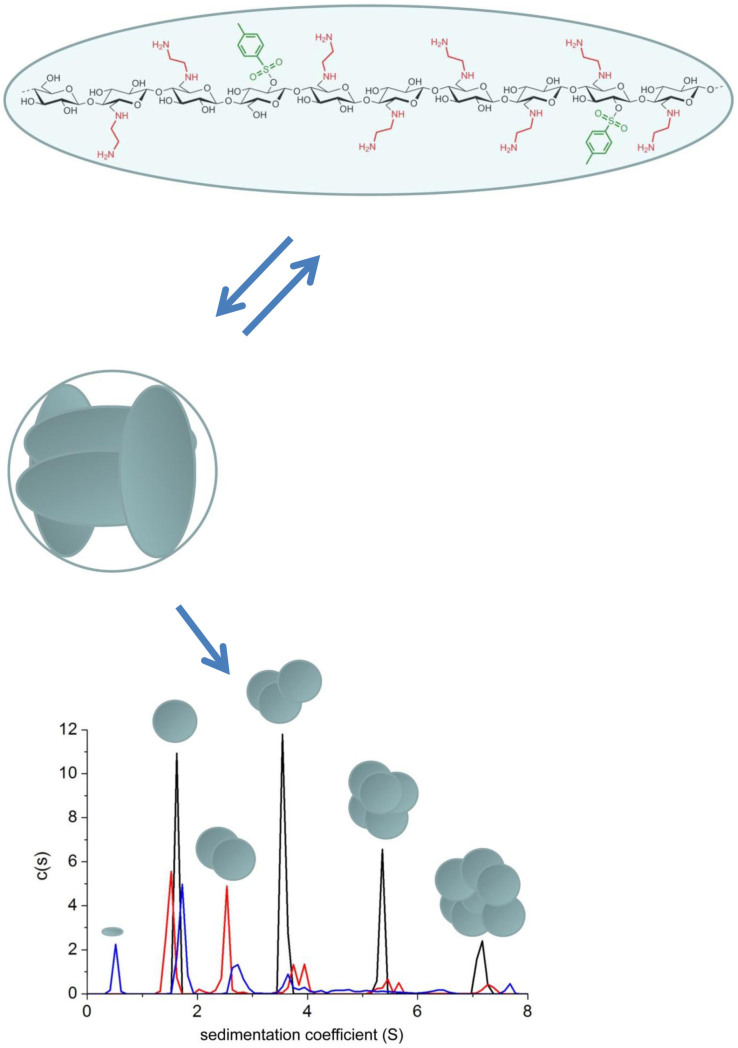
Reversible tetramerisation and further higher-order association of the polysaccharide 6-deoxy-6-(*ω*-aminoethyl)aminocellulose^2^ AEA-1. Top: Monomer unit of degree of polymerization ~10, degree of substitution at C-6 DS_Amine_ = 0.83 and degree of substitution at C-2 of tosyl residues DS_Tosyl_ = 0.2, yielding an M ~ 3250 Da and s ~ 0.5 S. Middle: Assembly into tetramers with M ~ 13000 Da and s ~ 1.7 S. Lower: Sedimentation coefficient distribution for AEA-1 at different concentrations 2.0 mg/ml (black), 1.0 mg/ml (red) and 0.75 mg/ml (blue). Based on the s ~ M^2/3^ scaling relationship the super-monomers associate into super-trimers, super-hexamers and super-9-mers with evidence also for some super-dimers, although the latter were not evident at the highest loading concentration. The proportion of the super-monomers drops relative to the higher order species showing partial reversibility even with the higher-order association.

1. The molecular mass of the monomer is ≈3250 Da (sedimentation coefficient, s ≈ 0.5 S). This corresponds to an average degree of polymerization of ~10 residues (Figure 2a).
2. These monomers reversibly associate through hydrogen bond interactions between the backbone –OH and –H residues as well as between the –NH_2_ residues reinforced by hydrophobic interactions between the tosyl residues to give a tetramer arranged together into a compact globular conformation of M ≈ 13000 Da and s ≈ 1.7 S (Figure 2b).
3. These globular tetramers (or “super-monomers”) further associate (partially reversibly) into higher order structures (Figure 2c) in a step-wise fashion (super-monomer, super-trimer, super-hexamer, super-9-mer (with some evidence also of some super-dimer) which themselves are consistent with an s ~ M^2/3^ rule, valid only for globular types of structures^10^ – this indicates that the assembly yields structures which are also globular in nature. This is consistent with the behaviour of many globular proteins, but has not heretofore been observed for a carbohydrate. The specific scaling rule that AEA-1 follows appears to be s_i_ ~ i^2/3^ where i is the order of the oligomer, with i = 1,3,6,9… This has been seen for other aminocelluloses^1^, but where i = 1,2,3,4….

The two stage assembly (Figure 2) – a full reversible tetramerisation stage followed by further assembly of the tetramers into larger assemblies - mirrors for example the tetramerisation of hemoglobin followed by assembly though hydrophobic interaction of these tetramers into higher order structures in sickle cell deoxyhemoglobin^11^.

## Discussion

Besides the functional significance of the associative processes of these molecules in their use as self-assembling films/matrices for loading with bioactive materials using NH_2_-reactive bifunctional reagents^2^, the observation of complete reversibility within these structures challenges the traditional perceptions of what is seen as “protein behaviour” and “carbohydrate behaviour”, opening exciting new opportunities for biomedical and commercial exploitation. These substances are already known to produce controllable self-assembling films for enzyme immobilisation and other biotechnological applications^2^, although the observation and understanding now of the existence of a fully reversible protein-like reversible tetramerisation in a subset of these substances opens up new possibilities for manipulation, particularly as their films are transparent and able to carry charge. The importance of self-assembly as a phenomenon in structural biology is evident: both from the point of view of our understanding of cell mechanics and possible defects of clinical consequence and our potential ability to devise strategies for the construction of novel materials^12^,^13^. The large self-assembling cationic structures render them as possibilities for mimicking the properties of histones and use as condensing or packing agents in DNA-based therapies^14^.

In adding carbohydrate to the list of fully reversible self-interacting structures we open up possibilities under both of these headings. Our use of the term “protein like” covers the step-wise oligomeristion consistent with a globular conformation for the species present, and with a fully reversible tetramerisation. However, whether or not the assembly process has features which parallel those recently proposed for proteins^15^ awaits further structural studies, including for example, whether a unique interface is formed between polymer chain, whether the each polymer oligomer adopts a well-defined structure.

Besides sickle cell hemoglobin, another example of the huge consequences associative or aggregative phenomena can cause in proteins is with amyloid assembly, although such processes do not appear reversible^16^,^17^,^18^,^19^,^20^.

It important to point out that although all 6-deoxy-6-(*ω*-aminoalkyl) aminocelluloses studied so far have shown stepwise oligomerisation behaviour^1^, the fully reversible initial stage has thus far only been seen in AEA-1. Another aminocellulose studied – M902TODA - an 6-deoxy-6-(13-amino-4,7,10-trioxa-tridecane-amino) (ATOTA) cellulose - for example does not show the full reversibility^21^.

It is also worth pointing out that chain association involving carbohydrates is not new. For example large micron size irreversible association of chitosans – considered for use as mucoadhesives for use in oral and nasal drug administration – has also been demonstrated by analytical ultracentrifugation and associated imaging techniques - with mucus glycoproteins^22^. Polysaccharides modified with hydrophobic groups have been shown to form nanoparticles^23^ and polysaccharides have been used as composite materials for microencapsulation^24^. At the other end of the scale a very weak reversible dimerisation has been observed in arabinoxylans on the basis of an increase in the sedimentation coefficient with concentration^25^. More significantly, chain dimerisation and aggregation of polysaccharides such as carrageenans^26^,^27^, xanthans^28^, alginates and pectins^29^ has also been claimed, but these processes largely depend on either thermal treatment, or the addition of divalent cations, and are not truly reversible in the thermodynamic sense (i.e. simply by adjusting the total concentration).

The tetramerisation stage observed in this study is the first genuine example of a non-weak fully reversible self-association in carbohydrates. Knowledge of the precise chemical nature of the carbohydrate interaction is likely to have a major relevance to the modes of interaction between glycoproteins, ubiquitously present across the range of cellular structures, and in gaining a greater ability to delineate aggregation phenomena from genuine assembly processes at the cellular level and below^30^.

## Methods

### Sample preparation

Preparation of the **6-deoxy-6-(*ω*-aminoalkyl)aminocellulose** amino-cellulose AEA-1 was as described earlier^1^. Tosyl cellulose was prepared according to Rahn *et al*. in DMA/LiCl/triethylamine^31^. NMR spectra were acquired on a Bruker Advance 250 MHz or 400 MHz spectrometer with 16 scans for ^1^H NMR and up to 21,000 scans for ^13^C NMR measurements with a sample concentration of 60 mg/mL. A CHNS 932 Analyzer (Leco) was used for elemental analysis.

### Sedimentation velocity analyses

Sedimentation velocity experiments were performed using an Optima XL-I analytical ultracentrifuge (Beckman Instruments, Palo Alto, USA). Phosphate buffered saline solutions (0.1 M) containing 0.125 to 2.0 mg/mL amino cellulose and reference solvent (0.1 M PBS buffer, 400 μL) were injected into solvent and sample channels of 12 mm carbon filled centerpieces and loaded into an eight-hole titanium rotor. Samples were run at 20.0°C with a rotor speed of 45,000 rpm. Concentration profiles at fixed time intervals in the analytical ultracentrifuge cell were registered using the Rayleigh interference optical system. Standard Fourier transform software converted the fringe profiles into plots of fringe displacement, ‘j' (relative to the meniscus) versus radial position, ‘r'. The data were analysed using the least squares c(s) method of P. Schuck and included in the SEDFIT program^4^,^32^. This software, based on the numerical solution to the Lamm equation for each sedimenting species, generates an apparent distribution of sedimentation coefficients, c(s) versus the sedimentation coefficient s (in Svedberg units, S, where 1 S = 10^−13^ seconds), correcting for the effects of diffusion.

### Sedimentation equilibrium analyses

Sedimentation equilibrium (SE) analyses^6^,^7^,^8^,^9^,^10^ were performed using a short solution column (~80 μL), at a low concentration (0.4 mg/mL) but also at high rotor speed of 40,000 rpm. The experiments were performed at this speed and for 36 hours, by which time oligomers of size >2 S would be almost completely pelleted. To further limit the contribution of dimer and also of the small amounts of lower sedimentation coefficient value components seen in c(s) analysis, only data from the two upper quartiles were accepted for analysis.

Molecular masses were evaluated as a function of radial position *r* in the ultracentrifuge cell using the routine MULTISIG^21^ based on the classical equation for a multi-component solute at sedimentation equilibrium^10^, with the equilibrium concentration distribution recorded using Rayleigh interference optics. The final distribution of concentration or fringe values (J_r_) w.r.t. radius (*r*) is given by 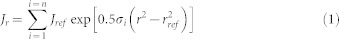
for a system of a large number (n) of components, where σ_i_ is the reduced flotational mass of the i-th component, and J_ref_ is a reference value at a radial position *r*_ref_. This reference position can be at any position in the cell: however the point averages yielded by MULTISIG will be referenced to whatever radial value is specified by the user. The parameter σ is defined by^6^

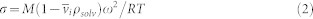
where M is the molecular mass of the solute (in Daltons or g/mol), R is the gas constant, T the temperature (°K), 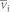 the partial specific volume of the solute component (ml/g), ρ_solv_ the density of the solvent and ω the angular velocity (radians/sec) of the rotor. The data set of J_r_ versus r is transformed into one of c_i_(σ_i_) versus σ_i_ at selected radial position, r in the cell by the routine MULTISIG^21^. By repetition of this fit at a sequence of defined radial positions using the routine MULTISIG_radius the various averages can then be evaluated as a function of r. The molecular mass moments are in all cases evaluated from: 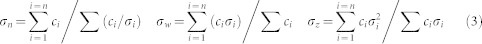
and hence M_n_(r), M_w_(r) and M_z_(r) obtained using equation [2]. MULTISIG may be compared to the classical least squares procedure of Roark and Yphantis^6^ except that the need for differentiation/double differentiation of the data is avoided, greatly reducing the noise level in final estimates and avoiding the need for pre-smoothing of raw data.

## Author Contributions

M.N. and T.H. were responsible for the synthesis and purification of the aminocelluloses. M.N. along with R.B.G. and D.T.B. were responsible for operating and running the analytical ultracentrifugation (AUC) experiments, A.J.R., M.N., G.G.A. and S.E.H. were responsible for the AUC analyses (sedimentation velocity and sedimentation equilibrium), S.E.H. and T.H. for writing the paper. All authors reviewed the manuscript.

## Supplementary Material

Supplementary Information Supplementary Information
