# Supplementary material for: Protein–like fully reversible tetramerisation and super-association of an aminocellulose
Source: Sci Rep. 2014 Jan 24;4:3861. doi: 10.1038/srep03861 (PMC3900928; doi:10.1038/srep03861)
Supplement: Supplementary Information [file srep03861-s1.doc]

# Protein –like fully reversible tetramerisation and super-association of an aminocellulose

# Melanie Nikolajski, Gary G. Adams, Richard Gillis, David Tabot Besong, Arthur J. Rowe, Thomas Heinze and Stephen E. Harding

**Supplementary Information**

An example of reversible associative behavior in protein systems from sedimentation equilibrium experiments in an analytical ultracentrifuge, the electron transfer flavoprotein ETF (Figure S1), and a classical example of non-associative behavior in a carbohydrate based polymer system (a mucin from a cystic fibrosis patient), Figure S2.


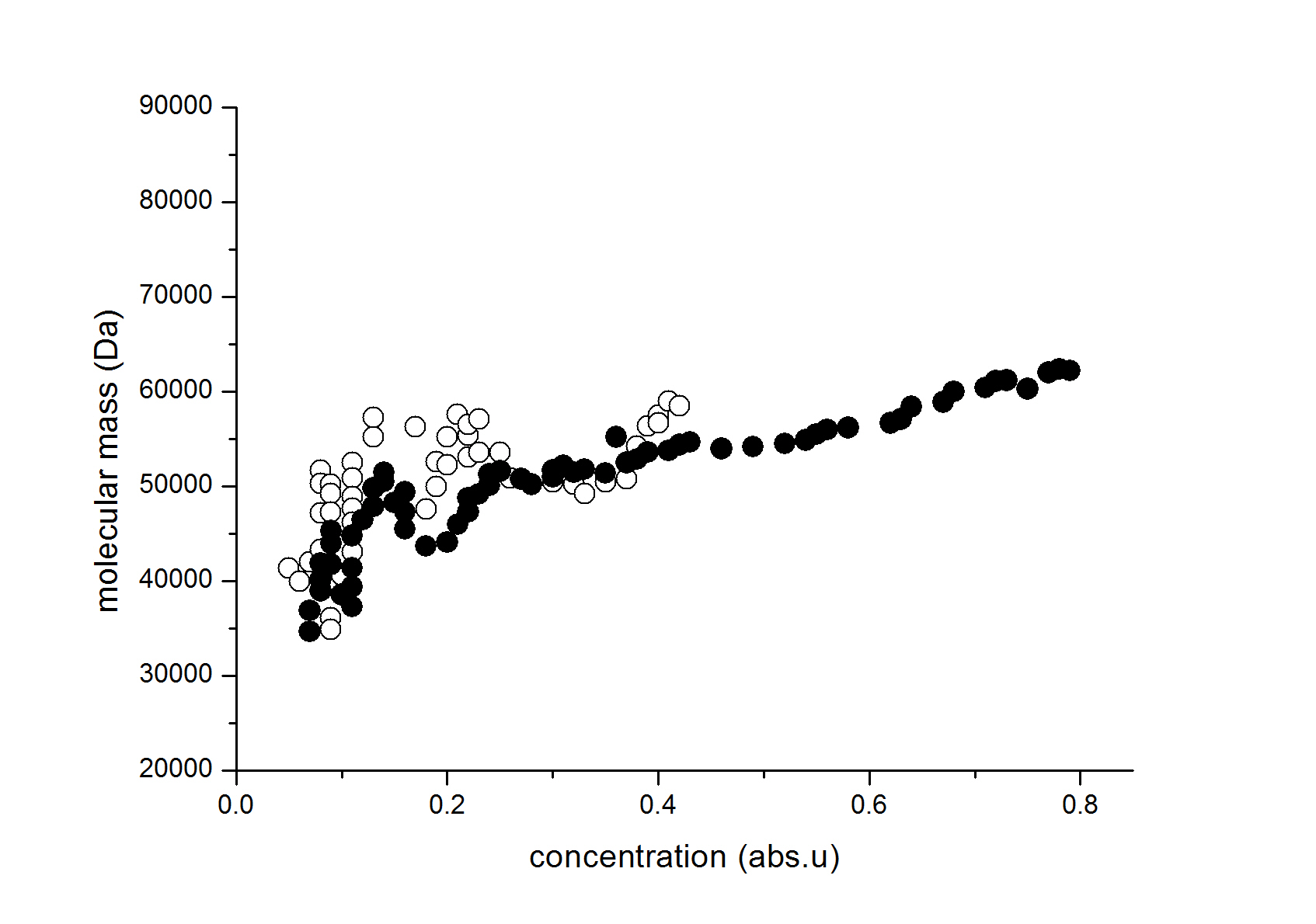


**Figure S1. Illustration of classical reversible self-association behaviour of a protein: electron transferring flavoprotein ETF.** Sedimentation equilibrium experiment in an analytical ultracentrifuge equipped with a uv-absorption optical system, rotor speed 17000 rpm and a temperature of 4^o^C. Solvent: potassium phosphate chloride buffer, pH=7.5, I=0.1. Plotted is the weight average molar mass as a function of local concentration (in expresed in absorbance units at a wavelength of 280nm in a 12mm optical path length cell) at various radial positions of the ultracentrifuge cell and for 2 different loading open circles: 0.4 mg/ml; circles: solid circles: 0.7 mg/ml. Despite the extra noise at low concentration (a feature of the absorption optical system) the points lie on the same overall curve. Reprinted, with permission, from ref S1. Other examples are given in for example Teller^S2^


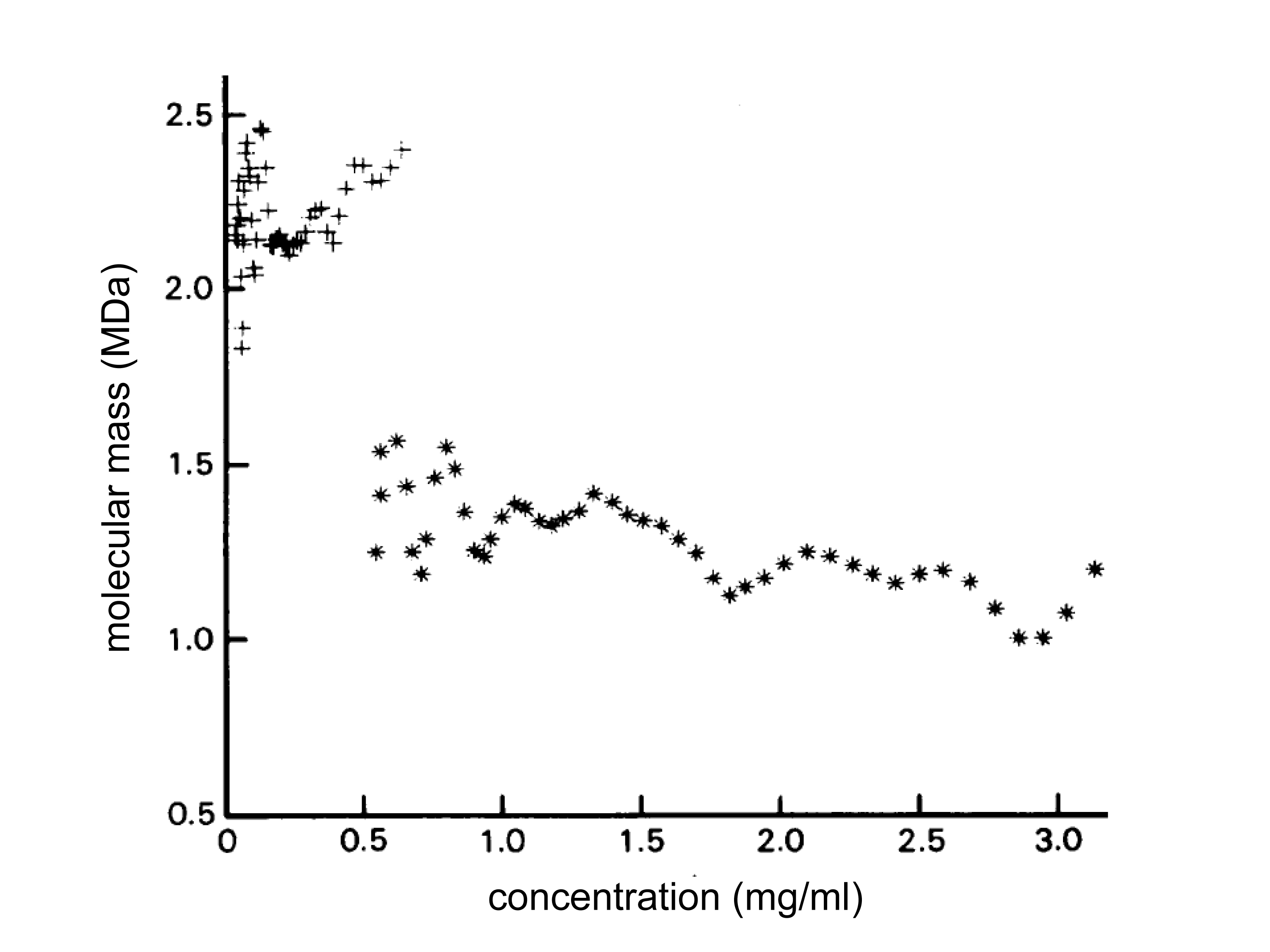


**Figure S2. A non reversibly associating system: a mucin (CF PHI) from the bronchial secretion of a patient with cystic fibrosis.** Sedimentation equilibrium experiment in an analytical ultracentrifuge equipped with Rayleigh interference optics, rotor speed 1967 rpm and a temperature of 20.0^o^C. Solvent: phosphare-chloride buffer containing 1M NaCl. Plot of weight average molar mass as a function of local concentration at various radial positions in the ultracentrifuge cell and for 2 different loading concentration: plus signs: 0.2 mg/ml; stars: 2.0 mg/ml. The two datasets clearly do not follow the same curve. Reprinted, with permission, from ref S3.

Both of the above cases – when compared with Figure 1 of the main text - also illustrate the enhanced precision of the currently employed analytical methods for defining interactions in sedimentation equilibrium.

**Supplementary References**

S1. Cölfen, H., Harding, S.E., Wilson, E.K., Scrutton, N.S. & Winzor, D.J., *European Biophys. J.* **25**, 411-416 (1997)

S2. Teller, D.C. *Meth. Enzymol.* **27D**, 346-441 (1973).

S3. Harding, S.E. *Biochem. J*. **219**, 1061-1064 (1984)
